# Cases of Neuralgia

**Published:** 1824-10

**Authors:** Benjamin Hutchinson


### Art. III.—*Cases of Neuralgia.* Communicated by Benjamin Hutchinson, Esq.

*To the Editors of the London Medical and Physical Journal.*

Gentlemen,—I have extracted from my case-book the following examples of Neuralgia, which may be acceptable to the numerous readers of your Journal.

I remain, Sir, your obedient servant,

BENJ. HUTCHINSON,
Fellow of the Royal College of Surgeons.

Miss Rebecca Holmes, of Normanton, near Southwell, a young lady, of a pale, leuco-phlegmatic complexion, between twenty and thirty years of age, and of a delicate constitution. My patient has been severely suffering, during the last three years, with intermitting pains in the right side of her face. She states that this painful affection began in the upper jaw, and extended in a very short time over the whole of the right side of the face to the temple. Several paroxysms occurred in the course of each day, and her rest at night was generally interrupted by the agony and beating of her temples. She was suffering most acutely when she first presented herself to my notice. She was, at all times since her first attack, unable to bear the least air to blow upon the parts affected; her bowels were constipated, her pulse feeble, and her tongue deeply furred. The pain extended also very frequently to the upper and lower lip, the left ala nasi, and to the superior bicuspid teeth.

The case assumed the true character and every accurately-marked symptom of a severe tic douloureux. Having but little dependence on any other mode of attacking this disease, I immediately began my assault with the same weapons which I had, on many former occasions, found eminently successful. A brisk cathartic or two of calomel and jalap preceded the use of the ferri subcarbonas; and in the course of six weeks her painful enemy was wholly subdued.

*Neuralgia Sciatica.*—Thomas Pearson, of Gonalston, in the county of Nottingham, fifty-six years of age, and of a robust constitution, the subject of the following very severe case of Neuralgia Sciatica, began, in the month of October, 1822, to complain of most acute pains in his loins and right hip, frequently extending to the groin, pubis, and the whole of the thigh. He states that his complaints had, during many months prior to this severe attack, been holding out menaces of a very unpleasant nature; his knees, ankles, and almost the whole circle of his joints, giving him, in some degree, a fore-warning of what he was subsequently to expect. In October, he was deprived of the power of following his occupation as a day-labourer, by the excessive increase of pain in these parts. He describes his miseries by comparing them, in the first instance, to a creeping sensation in his right hip, extending down to the knee and leg, and occasionally shooting towards the pubis and groin. This sleepy, benumbing feeling was almost immediately converted into pain of the most acute and lancinating kind, especially when attempting to move the affected parts. These torments were not accompanied by any considerable constitutional fever. He was placed, by his master, under the care of two eminent physicians in Nottingham; who ineffectually endeavoured to give the poor sufferer relief by the usual means of bleeding, blistering, the bark, opiates, colchicum, guaiacum, &c. &c. After many months of acute suffering, I was consulted by the poor man's master on this very distressing case, and immediately commenced my treatment with an active mercurial purgative; after the due operation of which, he began with large doses of the subcarbonate of iron three times a-day,—viz. one drachm to each dose. At bed-time, he took a couple of pills containing a proportion of the narcotic vegetable extracts; and the emetic-tartar ointment was used twice a-day to the parts affected, by which a very copious crop of pustules was very speedily elicited. In the course of a very few days, a manifest change for the better had taken place; his pains were much mitigated, and he was soon enabled to leave his bed. He is now perfectly well, and has long resumed his employments as a day-labourer.

" *To B. Hutchinson, Esq.*

" *Nottingham; March 26th,* 1824.

" My dear Sir,—Assisted by Mrs. Lacey's memory, I am enabled to send you some few particulars of my tormenting case of tic douleureux. The first attack of this complaint occurred in the autumn of 1820, which I then imagined to proceed from some disease of my teeth, accompanied with the rheumatism in my face; and it continued to harrass me most violently during the whole of the winter and spring. I felt very little inconvenience from it during the summer; but, as the autumn advanced, the paroxysms became stronger, and continued to increase during the ensuing winter. In the spring of 1822, this painful affection of my face increased with such rapidity and violence, as to confine me to my room for the space of five or six weeks; at the commencement of which, my friends, with myself, deemed it necessary to call in my medical attendants, who immediately advised the application of leeches to my gums, as well as bleeding in the arm, with fomentations, liniments, internal medicines in great variety; and, lastly, the extraction of a tooth, which produced a very considerable increase of pain and inflammation in the face, so much so as to prevent me taking any thing but liquids for nearly a week. Not the most remote sensible benefit was derived from these means; but, as the temperature of the air became milder, the pain gradually abated, though not to so comfortable an extent as in the year preceding. At about the usual time it returned, but not with the same degree of severity as in the spring; when a friend advised me to try the tincture of gum guaiacum, aloes, and turpentine, to be taken in milk, which at first appeared to afford me some relief; but, after continuing it a very short time, it lost the desired effect, and this dreadful malady continued to increase, so much so as to unfit me for business nearly the whole of the winter and spring 1823; which so depressed my spirits, that I became divested of all hopes of ever being delivered from so lamentable a situation, when a friend accidentally calling upon me, and describing my case to him, he felt assured that my disease was the tic douloureux, and strongly urged my consulting you. I immediately adopted his advice, and the most happy results you are well aware of; and believe me it is my greatest desire to offer you every grateful acknowledgment in my power. My sensations and my present state of health are, indeed, such as I had never presumed to look forward to.

"Believe me, my dear Sir, to remain, your obliged and faithful servant,

"WILLIAM LACEY."

The case above related by my patient, a respectable grocer in Nottingham, was in every point a most deplorable one. His constitution, strength, spirits, and energies, were greatly impaired. The nerves more immediately affected were some twigs of the palatine branch of the superior maxillary going to the nose, the infra orbitar, the inferior maxillary, and the respiratory nerve of the face. I commenced my attack on this formidable and apparently invincible enemy, by two or three brisk doses of calomel, followed by a saline cathartic on the succeeding mornings. The subcarbonate of iron was then entered upon, in doses of one drachm three times a-day; and an anodyne, consisting of a small portion of the vegetable narcotics, was given in the evening. An amendment was happily experienced in a few days, and followed by the result which my patient has so pleasingly and gratefully expressed in his letter.

